# Economic burden of smoking-attributable diseases in China: A systematic review

**DOI:** 10.18332/tid/120102

**Published:** 2020-05-12

**Authors:** Lili Shi, Lumin Zhong, Yuyang Cai

**Affiliations:** 1 Xinhua Hospital, Shanghai Jiao Tong University School of Medicine, Shanghai, China; 2 School of Public Health, Shanghai Jiao Tong University School of Medicine, Shanghai, China; 3 Department of Epidemiology and Public Health, University College London, London, United Kingdom; 4 NHC Key Laboratory of Health Economics and Policy Research, Shandong University, Jinan, China

**Keywords:** smoking, systematic review, economic burden

## Abstract

**INTRODUCTION:**

This review aims to synthesise the studies on smoking-attributable burden of diseases in China to assess the economic burden of smoking and highlight the weakness in these studies to inform future studies.

**METHODS:**

A systematic search of studies on smoking-attributable burden of disease in seven databases was conducted in 2019 and studies were screened according to inclusion and exclusion criteria. The evaluation of studies was based on the seven key elements for burden of disease studies. Costs were converted into 2013 Renminbi (RMB), with 1000 RMB about 163 US$ in 2013, the year of the first search, using the Consumer Price Index and the then exchange rate.

**RESULTS:**

Twenty studies were identified that estimated the costs of smoking in China, ranging from 57.162 to 368.273 billion RMB in total. The largest proportion of direct costs was allocated to outpatient visits, accounting for 49.17–68.94% of the direct costs. Meanwhile, costs resulting from mortality constituted 64.52–98.82% of the indirect costs. In mainland China, the understanding of PAR% (ratio of population attributable risk and incidence in the total population) in studies is not consistent. Studies on the cost of passive smoking are lacking and the research method for diseases needs to be improved.

**CONCLUSIONS:**

Smoking-attributable diseases have exerted substantial direct and indirect economic burden on China. The methodologies for future studies should be improved. Hong Kong and Taiwan provide good examples for future research in mainland China and researchers there should use PAR% correctly. More studies on the burden of passive smoking should be conducted. We propose a combination of single and all-disease research methods, if data are sufficient.

## INTRODUCTION

Economic burden of diseases focuses on the financial loss of individuals, families, societies and governments due to morbidity, early death (mortality), and the economic resources spent related to the treatment of diseases. Usually, the economic burden of diseases can be divided into direct and indirect burdens. The former refers to the total cost of treatment of diseases directly by individuals, families and society (e.g. outpatient fees, hospitalization etc.). The latter refers to the loss of the present and future value of society and the family, i.e. the economic loss of work leave, as a result of illness, disability and early death^1^. China is the world’s largest consumer of tobacco^2^, and smoking has become a hot topic because of its serious health hazards. Within the scope of China (including Taiwan and Hong Kong), this systematic review aims to synthesize the studies on the economic burden of smoking-attributable diseases. The objectives are to assess the economic burden of smoking-attributable diseases in China, evaluate the methodologies of these studies and provide a reference for future economic evaluations^3^. This study systematically searched seven databases in Chinese and English, summarized and analyzed the literature on the economic burden of diseases attributed to smoking in China, assessed the economic burden of smoking-attributable diseases in China, summed up the characteristics and problems of current methodologies in these studies, and puts forward corresponding improvement suggestions.

## METHODS

### Search strategies

Regarding the Cochrane Handbook Version 5.1, we used a combination of free words and keywords search as our search strategy. The search string was of the form: [smoking(吸烟 ) ] AND [burden of disease(疾病负担;)]; each part containing the corresponding free words in Chinese and English (Table 1) with the free words and subject words connected by OR. The subject words in Chinese were taken from the Chinese medical thesaurus of CBM. The subject words in English were taken from MeSH. The free words in Chinese and English were the entry words under each subject word. Search fields included titles, summaries, and keywords. We manually retrieved the list of references.

**Table 1:** Inclusion and exclusion criteria for articles

Inclusion criteria	Exclusion criteria
1. The language is Chinese or English.	1. The study only involves a part of the direct economic burden of smoking, such as the cost of hospitalization.
2. The study is on active smoking and/or passive smoking.	2. The study only includes cost-effectiveness analysis, cost-utility analysis, cost-benefit analysis, and epidemiological studies using disability-adjusted life years as the result.
3. The purpose of the study is to estimate the economic burden of smoking in China (including Hong Kong, Macao and Taiwan), including the economic burden of direct and/or indirect diseases.	3. Guidelines, comments, proceedings, newspaper articles etc.
4. The study is on the economic burden of smoking attributable diseases in China from the perspective of family and society.	

In December 2019, we searched for articles using seven databases in Chinese and English. The Chinese databases were: The General Library of Chinese Academic Journals (CNKI), Chinese Biomedical Literature Database (CBM), Wan fang Data-academic Journal Full Text library, and Database for Chinese Technical Periodicals (VIP). The databases in English were: PubMed, Ovid EMBASE: Excerpta Medica, and the Cochrane Library. All searches in the databases did not limit the starting date of publication. The end date of publication was set at 30th November 2019.

### Inclusion and exclusion criteria

The selection of literature was conducted independently by two researchers based on the inclusion and exclusion criteria given in Table 1.

### Selection process

**Figure 1:**
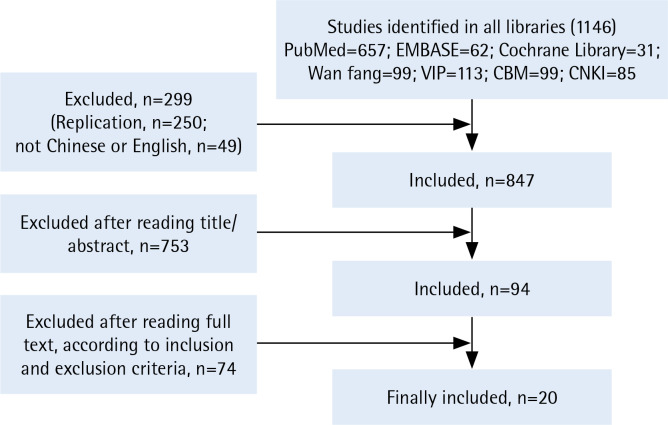
Prisma flow diagram of literature selection process

The researchers first screened and excluded the literature that did not meet the inclusion criteria by reading the title and summary. Then, a second round of screening was conducted by reading the full text of the literature, filtered from the initial screening. If differences were encountered, two researchers attempted to reach consensus through discussions or engaging the third researcher to make a final decision (Figure 1, and Supplementary file).

### Literature quality evaluation standard

Currently, there is no formal guide for the study of the economic burden of diseases. Therefore, the quality evaluation of the literature is based on the following seven key elements of research on the economic burden of diseases^4^: perspective, population, direct cost, indirect cost, discount rate, incremental cost, and sensitivity analysis. The research perspective can be the family, society, individual or government. Ideally, researchers should describe in detail the sources and demographic characteristics of the study population such as age and sex to improve comparability among different studies^5^. The research on the economic burden of diseases should include all the important and related costs from that particular research perspective, and clarify the source of the data used. The meaning of cost is the incremental cost caused by diseases. The discount rate should be used to convert the future value of money into its present value when estimating the economic loss of mortality. Sensitivity analysis is a method to check the stability of the results obtained under certain assumptions. Different values are assigned to the variables of uncertainty when estimating the economic burden of diseases, to determine the possible range of costs^6^.

### Data extraction and evaluation

The data extraction table was established with Epidata 3.1, including the basic information of literature, methodology used, the seven elements for literature evaluation, composition of the economic burden of disease attributable to smoking, and the corresponding cost estimate and proportion. Direct economic burden of disease is divided into the six components: hospitalization, outpatient, escort, transportation, medicine, and other. Indirect economic burden of disease is divided into three parts: morbidity, mortality, and other.

This study divides the disease study methods into three types: single-disease, disease-specific, and all-diseases. The single disease method is used to estimate the economic burden of a certain smoking-related disease; the disease-specific study is based on the ‘smoking-disease-economic burden’ as the framework to estimate the economic burden of each type of smoking-related disease, and uses the sum as the economic burden of disease attributable to smoking^7^; the all-diseases study was based on the ‘smoking-economic burden’ framework. By establishing an economic model, the economic burdens of smokers and non-smokers are compared, and the gap between the two is regarded as the economic burden of diseases attributable to smoking^8^. Also, using the consumer price index and exchange rate published in the China Statistical Yearbook, this study converted the estimated value of the cost of the economic burden attributable to smoking in different periods and different monetary units into 2013 RMB.

## RESULTS

### Overview of the included studies

A total of 1146 articles were retrieved. According to the inclusion and exclusion criteria, the literature was screened and 20 articles were finally included^9-28^: 16 studies in mainland China, 2 in Hong Kong, and 2 in Taiwan. By manually retrieving the list of references, no article was included. The earliest article was published in 1995^9^, and the latest article was in 2019^24^. The number of published studies has increased over time.

### The economic burden of smoking-attributable diseases

As shown in Table 2, the estimated value of the economic burden attributable to smoking in mainland China was between 57.162 billion RMB (36.31% direct and 63.69% indirect costs, respectively) and 368.273 billion RMB (58.02% direct and 41.98% indirect costs, respectively). The study by Li et al.^12^ has the highest estimated value because of the inclusion of the economic loss due to early death caused by passive smoking, fires caused by smoking, and the cost of pollution by the tobacco industry. Outside mainland China, the studies by McGhee et al.^26^ and Yang et al.^27^ show that the total costs of the economic burden of disease attributable to smoking in Hong Kong in 1998 and Taiwan in 2001 were 7.828 billion RMB (2013) and 20.15 billion RMB, respectively. In general, the outpatient cost is the largest part of the direct economic burden of disease attributable to smoking in China (excluding Hong Kong), taking up 49.17– 68.94% of direct disease economic burden, while the indirect disease economic burden is mainly caused by the economic loss of mortality, which accounts for about 64.52–98.82%.

**Table 2:** Estimates of cost-of-illness (million RMB) attribute to smoking in China, 2013

*Author and Year*	*Total economic burden of disease*	*Direct economic burden of disease*							*Indirect economic burden of disease*			
		*Total*	*Hospitalization*	*Outpatient visits*	*Care givers*	*Transportation*	*Drugs*	*Other*	*Total*	*Morbidity*	*Mortality*	*Other*
**Mainland**												
Jin et al. 1989^[9]^	76526.13	19619.19	-	-	-	-	-	-	56906.95	7265.32	49641.63	-
Jiang et al. 1998^[10]^	-	31037.55	7896.23	23141.32	-	-	-	-	-	-	-	-
Sung et al. 2000^[11]^	57162.10	20758.32	7430.64	10207.29	1390.54^a^	1390.54^a^	1729.85	-	36403.79	3072.16	33331.63	-
Li et al. 2005^[12]^	368273.41	213681.48	-	-	-	-	-	-	154591.93	646.59	137480.48	16464.87^b^
Yang et al. 2003^[13]^	135082.92	34404.84	5158.84	19569.60	1181.92^a^	1181.92^a^	8494.48	-	100678.08	1261.40	99416.68	-
Yang et al. 2008^[13]^	228329.96	52111.84	13153.52	35925.04	3033.28^a^	3033.28^a^	-	-	176218.12	2075.42	174142.70	-
Li et al. 2011^[14]^	-	84.01	-	-	-	-	-	-	-	-	-	-
Yao et al. 2011^[16]^	-	8006.61	4185.80	3820.81	-	-	-	-	-	-	-	-
Cai et al. 2010^[15]^	1268.21	1253.85	-	-	-	-	-	-	14.36	-	-	-
Yang et al. 2013^[17]^	-	-	-	-	-	-	-	-	289320.00	-	-	-
Chen et al. 2013^[19]^	1283.69	1118.17	-	-	-	-	-	-	165.52	56.94	57.66	50.92^d^
Yang et al. 2013^[20]^	-	96.19	-	-	-	-	-	-	-	-	-	-
Tang et al. 2013^[18]^	66.94	20.98	-	-	-	-	-	-	45.96	45.25	0.71	-
**Hong Kong**												
Leung et al. 1997^[25]^	-	28.72	23.85	4.87	-	-	-	-	-	-	-	-
McGhee et al. 1998^[26]^	7627.68	5159.90	2759.42	1031.98	1323.63	-	-	44.87^c^	2467.78	628.16	1951.79	-
**Taiwan**												
Yang et al. 2001^[27]^	20150.05	4481.03	1908.69	2572.34	-	-	-	-	-	-	15669.01	-
Sung et al. 2010^[28]^	12109.58	6005.38	2234.71	3770.67	-	-	-	-	6104.20	160.11	5944.09	-

a Combination of care-giving and transportation costs.

b Combination of tobacco industry pollution and fire caused by smoking costs.

c Combination of out-patient and hospitalization expenses attributable to passive smoking in children.

d Cost of passive smoking. RMB: Renminbi, 2013 exchange rate 1000 RMB about 163 US$.

### Literature quality evaluation

Among the included articles, 3 pointed out the research perspectives^10,15,16^, 9 articles did not but the perspective could be estimated according to the data sources in the articles^11,14,15,17,19,21-24^. One article had an unclear research perspective^9^. All 20 studies presented the demographic characteristics of the study population but only 4 described the sociodemographic characteristics of the study population^11,13-15^. Among the 14 articles that discussed the direct economic burden of diseases, 3 did not classify the cost^9,12,14^. Of the 10 studies that involved indirect economic loss^9-14,17-19,22^, 7 studies classified the indirect economic loss into loss due to morbidity and mortality^9-13,18,19^, and one study did not point out the components of indirect costs^22^. One study used the discount rate^12^. Incremental costs of smoking were estimated by population attributable risk (PAR%) in all articles but one^17^. Also, a sensitivity analysis using relative risk, smoking rate and the discount rate were carried out in 6 articles^11-16^.

### Evaluation of methodologies

As shown in Table 3, seventeen studies used the disease-specific research method from the perspective of society to estimate the economic burden of diseases attributable to smoking. Among the 16 included studies from mainland China, 14 studies used the disease-specific methodology and 2 used the single-disease methodology; most of them are clear about their research perspectives but one; only one study used the family perspective; 9 studies focused on active smoking and only 2 on passive smoking; 9 studies looked at both direct and indirect economic burden and 3 only looked at indirect economic burden. Seven studies set the scope of both active and passive smoking, among which 3 are worth noting. The study by Li et al.^12^ involved passive smoking but only calculated the economic loss of mortality caused by passive smoking. The study by another group, Li et al.^14^, limited the estimated value to the economic burden of hypertension caused by active and passive smoking in Luoping County, Yunnan province. Leung et al.^25^, using data from a birth cohort study of newborns in Hong Kong from 1997–1998, estimated the outpatient and hospitalization fees generated by exposure to secondhand smoke (SHS), also known as environmental tobacco smoke (ETS), during pregnancy.

**Table 3:** Methodology of included economic burden of diseases attributable to smoking studies in China

*Author and Year*	*Methodology*	*Research perspective*	*Research scope*	
			*Active/passive smoking*	*Direct/indirect economic burden*
**Mainland China**				
Jin et al. 1995^[9]^	Disease-specific	Unknown	Active	Direct+indirect
Jiang et al. 2000^[10]^	Disease-specific	Society	Active	Direct
Sung et al. 2006^[11]^	Disease-specific	Society	Active	Direct+indirect
Li et al. 2008^[12]^	Disease-specific	Society	Active+Passive	Direct+indirect
Yang et al. 2011^[13]^	Disease-specific	Society	Active	Direct+indirect
Li et al. 2013^[14]^	Single disease	Family	Active+Passive	Direct
Cai et al. 2014^[15]^	Disease-specific	Society	Active+Passive	Direct+indirect
Yao et al. 2015^[16]^	Disease-specific	Society	Passive	Direct
Yang et al. 2015^[17]^	Disease-specific	Society	Active	Indirect
Tang et al. 2016^[18]^	Disease-specific	Society	Active+Passive	Direct+indirect
Chen et al. 2016^[19]^	Disease-specific	Society	Active+Passive	Direct+indirect
Yang et al. 2017^[20]^	Disease-specific	Society	Active	Direct
Qi et al. 2018^[21]^	Disease-specific	Society	Active	Direct+indirect
Ma et al. 2018^[22]^	Disease-specific	Society	Active	Indirect
Wang et al. 2019^[23]^	Single disease	Society	Passive	Indirect
Fan et al. 2018^[24]^	Disease-specific	Society	Active	Indirect
**Hong Kong**				
Leung et al. 2003^[25]^	All diseases	Society	Passive	Direct
McGhee et al. 2006^[26]^	Disease-specific	Society	Active+Passive	Direct+indirect
**Taiwan**				
Yang et al. 2005^[27]^	Disease-specific	Society	Active	Direct+indirect
Sung et al. 2014^[28]^	Disease-specific	Society	Active+Passive	Direct+indirect

## DISCUSSION

This is the first study to synthesize studies on the economic burden of smoking-attributable diseases in China. By putting together estimates from previous studies, the scale of economic loss attributable to smoking was drawn in this systematic review, but there is a potential risk of biases.

### Confidence in cumulative evidence

Despite the declining smoking rate in the 20 years from 1989 to 2008^12^, the economic burden of smoking has increased from 76.526 billion RMB (2013) in 1989 to 228.33 billion RMB (2013) in 2008. This may be related to the lagging relationship between smoking and health outcomes and the rise in medical costs. The studies by Sung et al.^11^ and Yang et al.^13^ showed that the proportion of direct medical costs attributable to smoking in mainland China is 3–3.1% of the total national health expenditure, which is lower than that of India at 4.7%^29^ and US at 6–8%^30^, suggesting that the estimated values in the related studies may be lower than the actual values.

### Risk of bias assessment

First, the overall burden may be underestimated because only 56% of the studies calculated both direct and indirect economic burden, and most studies used known PAR% to calculate the smoking-attributable burden of diseases, however, choosing PAR% from past studies may underestimate actual burden contemporarily^10^. Second, only one study adopted the family perspective so it is hard to know about the economic burden of smoking-attributable diseases on Chinese families. Third, the economic burden of passive smoking can be a serious underestimation. For example, the study by Li et al.^12^ calculates the economic cost of just a few diseases including childhood asthma, breast and uterus cancer, due to active smoking but not infant malnutrition and other diseases, which can lead to serious underestimation. A recent study calculated the population attributable fraction (PAF) and disability-adjusted life years (DALY) for stroke due to passive smoking but did not give any actual cost^23^. Passive smokers account for 52.2% of China’s total population^31^, but only 2 studies focused on economic loss due to passive smoking (SHS).

### Comparisons with studies in Hong Kong and Taiwan

Hong Kong has especially focused on the direct economic burden of secondhand smoke (SHS). The study by Leung et al.^25^ examined the direct economic loss in association with SHS among Chinese infants using data from a birth cohort study. The exposures included a variety of types of SHS such as exposure to smoking during pregnancy and postnatal paternal smoking. The outcome measures were odds ratios of doctor consultations/hospitalizations. They also calculated PAR% by each risk factor. They calculated the total extra cost attributable to each SHS exposure and by adding up each attributable cost, they estimated the total direct cost for the 1997 birth cohort to be $39.4 million, composed of $28.9 million for hospitalizations and $10.5 million for outpatient visits. The study by McGhee et al.^26^ estimated direct and indirect tobacco-related burden of diseases, including the cost from passive smoking. The outcome measures were odds ratios of mortality of different causes (including lung cancer, liver cancer, COPD, ischaemic heart disease, stroke etc.), hospital visits, emergency and GP visits, use of nursing and domestic help, and lost time from work during productive years. In conclusion, the total additional cost in 1998 in Hong Kong was $532 million for active smoking and $156 million for passive smoking, with 23% of the total disease burden due to smoking caused by passive smoking.

The study of SHS in Hong Kong is a good example of studying the direct economic burden of secondhand smoke. To conduct similar studies in mainland China, researchers would require data from the datasets such as the birth cohort, which includes the type of SHS exposure, types of illness, numbers of hospital consultations, and all kinds of potential confounding factors such as breastfeeding history, demographic, and behavioural variables. The collection of this information would need the cooperation of parents. Measures should be taken to avoid possible recall bias.

Taiwan has also provided a good example of secondhand smoke (SHS) research methods. Yang et al.^27^ in Taiwan estimated the cost of smoking and SHS using SAF (smoking-attributable fraction). The study used the prevalence-based method (smokingattributable fraction) to estimate the direct and indirect costs of smoking and secondhand smoke (SHS) of the population aged ≥35 years in 2011 with the data from the 2010 population census. They calculated the SAF and SAF(SHS) for each part of the cost by each disease, gender, and age group. The smoking-attributable inpatient/outpatient expenditures were calculated by multiplying the SAF by the total annual inpatient/outpatient expenditure of each subgroup. The SHS-attributable inpatient/outpatient expenditures were calculated by multiplying the SAF(SHS) by the total annual inpatient/outpatient expenditure for each disease among non-smokers.

### Implications for future studies

In the future, to make the research results more comprehensive and comparable, researchers should work on their research in the following aspects. First, the application and understanding of PAR% in the calculation of smoking-attributable burden of diseases should be unified or stated more clearly in studies in mainland China. Jiang et al.^10^ used PAR% from past studies to calculate the economic burden. In the study by Wang et al.^23^, the smoking-attributable relative risk of stroke was chosen from the literature to calculate PAR%. These approaches may lead to underestimation of the smoking-attributable burden of diseases, while the heterogeneity of calculation methods cause incomparability across studies. The study by Leung et al.^25^ calculated new PAR% of costs of medical services in Hong Kong using collected data, which is a much more reliable approach. The study by Rice et al.^32^ shows that on average PAR% calculated by the relative risk of death is 3.8% lower than that calculated by the relative risk of medical service utilization. Second, we suggest conducting more studies on the cost of passive smoking, especially in mainland China as they are lacking at the moment. As the study from Hong Kong shows, the burden from passive smoking was almost a quarter of the total cost and given the fact that China has a higher rate of smoking among men, the economic burden from passive smoking could be even higher. Third, the disease research method should be improved. Compared with the all-disease research method, the estimation process of the disease-specific research method is rather simple and less data-demanding. But as the disease-specific method is limited to only a few types of smoking-related diseases, the reported economic burden of diseases from this kind of study may be underestimated^33^. At this stage, studies on the economic burden of diseases caused by smoking in China is still largely based on the disease-specific research method, and most only involve the major diseases such as malignant tumours, and cardiovascular system and respiratory system disease. The study by Yang et al.^33^ suggests that the costs of kidney disease accounts for 21.28% of the direct medical costs attributable to smoking, and is an important part of the total economic burden of diseases attributable to smoking. On the other hand, the all-disease method uses economic models to express the relationship between smoking and the economic burden of disease while controlling for the influence of socioeconomic and cultural factors. However, Warner et al.^30^ claim that this research method ignores the relevant intensity of smoking and disease, and can easily lead to overestimated results. Therefore, for future research, we propose the use of both single and all-disease research methods in one study, if data are sufficient.

## CONCLUSIONS

In general, smoking, as a major public health problem, has brought a huge economic burden to China, but the estimates from studies on the economic burden of smoking-attributable diseases result in underestimation by focusing on only some of the diseases or just on active smoking. Furthermore, studies on economic burden due to passive smoking in mainland China are lacking, and the methodologies and quality of studies on the economic burden of smoking-attributable diseases needs to be improved.
